# Rapamycin and trametinib: a rational combination for treatment of NSCLC

**DOI:** 10.7150/ijbs.62752

**Published:** 2021-07-25

**Authors:** Chao-Yue Sun, Yi-Zhuo Li, Di Cao, Yu-Feng Zhou, Mei-Yin Zhang, Hui-Yun Wang

**Affiliations:** 1 State Key Laboratory of Oncology in South China, Collaborative Innovation Center for Cancer Medicine, Sun Yat-Sen University Cancer Center, 651 Dongfeng East Road, Guangzhou, China 510060.; 2 Department of Medical Imaging, Sun Yat-Sen University Cancer Center, 651 Dongfeng East Road, Guangzhou, China 510060.

**Keywords:** Rapamycin, Trametinib, Synergy, Lung cancer, ERK, mTOR

## Abstract

Mammalian target of rapamycin (mTOR) is one of the most commonly activated pathways in human cancers, including lung cancer. Targeting mTOR with molecule inhibitors is considered as a useful therapeutic strategy. However, the results obtained from the clinical trials with the inhibitors so far have not met the original expectations, largely because of the drug resistance. Thus, combined or multiple drug therapy can bring about more favorable clinical outcomes. Here, we found that activation of ERK pathway was responsible for rapamycin drug resistance in non-small-cell lung cancer (NSCLC) cells. Accordingly, rapamycin-resistant NSCLC cells were more sensitive to ERK inhibitor (ERKi), trametinib, and in turn, trametinib-resistant NSCLC cells were also susceptible to rapamycin. Combining rapamycin with trametinib led to a potent synergistic antitumor efficacy, which induced G1-phase cycle arrest and apoptosis. In addition, rapamycin synergized with another ERKi, MEK162, and in turn, trametinib synergized with other mTORi, Torin1 and OSI-027. Mechanistically, rapamycin in combination with trametinib resulted in a greater decrease of phosphorylation of AKT, ERK, mTOR and 4EBP1. In xenograft mouse model, co-administration of rapamycin and trametinib caused a substantial suppression in tumor growth without obvious drug toxicity. Overall, our study identifies a reasonable combined strategy for treatment of NSCLC.

## Introduction

Lung cancer remains the leading cause of cancer-related death in men and women worldwide, which kills more than 1.7 million people every year 1, 2. Non-small cell lung cancer (NSCLC) accounts for about 80-85% of all lung cancer cases, and most newly diagnosed patients with NSCLC are in advanced-stage, with a dismal 18.6% 5-year survival rate 3. Options for treatment of NSCLC patients are limited after failure of standard first-line therapy (including radical local therapy: surgery or radiotherapy, and systemic chemotherapy). Although great strides in the development of molecules targeting tyrosine kinases or immune checkpoints have been made, drug resistance to these inhibitors rapidly develops. Thus, there is an urgent need for new combination strategies to provide better treatment outcomes for NSCLC patients in the future.

Mammalian target of rapamycin (mTOR) pathway has been recognized as an important player in regulation of cell proliferation and metabolism 4. Numerous reports indicate that mTOR is one of the most frequently activated pathways in human cancers 5, thus it attracts considerable interest as an essential target for cancer treatment 6. Several mTOR inhibitors (mTORi) are approved for the treatment of many types of cancer 7. However, acquired resistance of mTORi remains a major treatment challenge. For example, mTORi can elicit a feedback-dependent activation pathway, such as AKT, leading to mTORi drug resistance 8. Among these inhibitors, the natural compound, rapamycin is the first generation of mTORi that has been approved by FDA as a potent immunosuppressant after kidney transplants, and to treat lymphangioleiomyomatosis 7, 9. Although rapamycin is used as an antineoplastic agent in certain types of cancer, its clinical outcomes are mostly disappointing 10. To circumvent this issue, its analogs have been developed, such as temsirolimus and everolimus 6. However, these derivatives have not displayed clinical success. Thus, it is important to understand the possible mechanisms of drug resistance of mTORi, and develop new strategy to improve the clinical benefits of mTORi.

The RAS/RAF/MEK/ERK pathway (ERK) is an evolutionary conserved signaling cascade that involves in various cellular processes 11. The importance of ERK in cancer cell biology has been well documented 12. ERK is aberrantly activated in more than 30% of human cancers 13. In addition, numerous standard treatments, including, chemotherapy, radiotherapy, targeted agents and immunotherapy can lead to ERK activation that acts as a cell survival mechanism, which confers resistance to the treatments 14, 15. Interestingly, phospho-ERK has been identified as a potent biomarker for combination treatment of sorafenib with ERK inhibitors in liver cancer 16. Inhibition of ERK, therefore, is a promising therapeutic strategy for human cancers. Trametinib, a ERK inhibitor (ERKi), has been approved by the FDA as a monotherapy or in combination with dabrafenib, for the treatment of patients with BRAF V600E/K^+^ unresectable or metastatic melanoma 17. However, trametinib and other ERKi also can induce activation of pro-survival feedback mechanisms, such as AKT and autophagy 18, 19. Thus, combined approaches of ERKi and other agents to counteract these feedbacks are promising strategy for cancer treatment.

In this study, we sought evidence for the rational combination of rapamycin with trametinib in NSCLC cells. We demonstrated that, activation of ERK was observed in rapamycin-resistant or single-dose of rapamycin-treated cells. Accordingly, trametinib treatment overcame drug resistance of rapamycin, and synergized with rapamycin to inhibit tumor growth *in vitro* and *in vivo* . Thus, combined rapamycin and trametinib is a high therapeutic priority for NSCLC.

## Materials and Methods

### Reagents

Rapamycin and binimetinib (MEK162) were purchased from Selleck Chemicals (Houston, USA). Trametinib, Torin1 and OSI-027 were obtained from TargetMol (Boston, USA). All drugs were dissolved in DMSO to yield 10 mM stock and stored in the -20 °C.

### Cell culture and selection of drug resistant cells

Human NSCLC cell lines, A549, PC9, H1650 and liver cancer cell line, Huh7, were obtained from the State Key Laboratory of Oncology in South China. All NSCLC cells were cultured in RPMI 1640 medium, while Huh7 cells were cultured DMEM medium containing 10% fetal bovine serum (FBS, Gibco, US) at 37 °C in a humidified incubator with an atmosphere of 5% CO_2_. NSCLC cell lines resistant to rapamycin or trametinib were generated by exposing the parental A549 and H1650 to a high dose of rapamycin (4μM) or trametinib (100nM) for 3 months of continuous drug exposure 5. Then, rapamycin or trametinib-resistant cells (A549-R, H1650-R, A549-T, H1650-T) were obtained.

### Measurement of cell viability and cell death

The cell viability was measured using a Cell Counting Kit-8 (CCK-8, Beyotime, China) following the manufacturer's protocol. Briefly, cells were seeded in 96-well plates at 40,000 cells per well and treated with rapamycin alone, or trametinib alone, or in combination for 48 hours. Then, cells were subjected to CCK8 solution. After 3 hours of incubation, the cell numbers were measured using a spectrophotometer at 450 nm. For drug interaction analysis, combination index (CI) was analysed by the Chou-Talalay method using the CompuSyn software. The fraction affected (Fa) was calculated by the percent growth inhibition, and 0.9 < CI < 1.1 indicates additive effect, CI < 0.9 indicates synergism.

Cell death was analyzed by staining SYTOX Green (KeyGEN, China) according to the manufacturer's instructions. Cells were seeded in 6-well plates and treated with rapamycin alone, or trametinib alone, or in combination for 24 hours. After treatment, cells were washed by PBS and incubated in SYTOX Green solution for 10 minutes. Then, the stain medium was replaced with PBS, followed by using microscopy to detect dead cells.

### Cell cycle and apoptosis analysis

Cells were seeded in 6-well plates and treated with rapamycin alone, or trametinib alone, or in combination for 48 hours. Then, cells were washed in PBS and collected by centrifugation. For cell cycle, cells were resuspended by DNA staining solution and 10 μL permeabilization solution (Multi Sciences), and incubated for 30 minutes. Samples were analyzed by flow cytometer (BD, USA). For detection of apoptosis, cells were resuspended with Binding buffer, and stained with propidium iodide (PI) and Annexin V-AF647 double staining (Yishan, China) for 5 minutes, according to the manufacturer's instructions. Analysis was performed on flow cytometer (BD, USA).

### Western blot analysis

Cells were washed with PBS, and lysed on ice with RIPA lysis buffer (Beyotime, China) containing protease and phosphatase inhibitors (Pierce Chemical). Protein concentrations were determined by the BCA Kit (Thermo Scientific) according to manufacturer's instructions. The prepared lysates were applied to SDS-PAGE and then transferred to PVDF membrane (Millipore, USA). After 1h of blocking with 5% skim milk, the membrane was immunoblotted with primary antibodies for GAPDH (CST, #5174), 4EBP1 (CST, #9644), p-4EBP1 (CST, #2855), mTOR (CST, #2972), p-mTOR (CST, #5536), S6K (CST, #2708), P-S6K (CST, #9234), ERK (CST, #4695), p-ERK (CST, #4370), AKT (CST, #4691), p-AKT (CST, #4060), PARP (CST, #9542), cleaved PARP (CST, #5625), CDK2 (CST, #2546), CDK4 (CST, #3136), c-myc (Abcam, #ab32072). After an hour incubation with secondary antibodies, bands were visualized with enhanced chemiluminescence substrates (Millipore) and images were processed by ImageLab Software.

### Kaplan-Meier Plotter analysis

Kaplan-Meier plotter (http://kmplot.com/analysis/) was used to assess the prognostic value of ERK, AKT, c-myc, mTOR and 4EBP1 in lung cancer patients. These genes mRNA levels in lung cancer patients were obtained using this web that provides patient survival information. Patients are divided into two groups, based on median mRNA expression, and validated by a Kaplan-Meier survival curve, with the hazard ratio (HR), 95% confidence intervals (CIs) and p values.

### *In vivo* tumor xenograft studies

All mouse experiments complied with protocols approved by the Animal Care and Use Committee of Sun Yat-Sen University Cancer Center (SYSUCC, protocol ID: protocol ID: L102012020020E). Female BALB/c nude mice at 4-6 weeks of age were purchased from Guangdong Medical Laboratory Animal Center (Foshan, China). PC9 cells (4×10^6^) were subcutaneously injected into the left flank of mice. Once tumor volume reached an average of 150 to 200 mm^3^, the mice were randomized to four treatment groups: control (vehicle); rapamycin (1mg/kg, i.p.); trametinib (0.5mg/kg, i.g.); rapamycin + trametinib. Trametinib was dissolved in DMSO and diluted in water with 0.5% carboxymethylcellulose and 0.2% Tween-80 (Sigma), and rapamycin was dissolved in DMSO and then diluted in saline solution. All drugs were administered every day, continuously for 9 days. Tumor volume was measured by calipers every day and calculated by the following equation: V=length × width^2^/2. After treatment, mice were sacrificed, and the tumor tissues were resected, weighed and used for further analysis.

### Immunohistochemistry

Immunohistochemistry assays were performed as previously described. Tissues were fixed overnight in paraformaldehyde, and then paraffin-embedded, sectioned and deparaffinized using xylene and graded ethanol series. The sections were pre-treated using heat mediated antigen retrieval with Tris-EDTA buffer and blocked with 5% BSA. Then the sections were immunostained overnight with PCNA (CST, #9542, 1:2000). After being stained with a secondary antibody (KeyGEN, China), DAB chromogen solution was used to allow for proper brown color development, followed by the nuclear counter-staining using hematoxylin. Images were captured at 200 or 400 × magnification using a microscopy.

### Statistical analyses

Statistical analysis was performed using the GraphPad Prism 7. All data represent as mean ± SD from at least three independent experiments. Comparisons between groups were performed using Student's t tests, and one-way ANOVA was used to compare values between multiple groups. P values less than 0.05 were considered significant.

## Results

### ERK inhibition circumvents resistance to mTOR blockade

**Figure 1:**
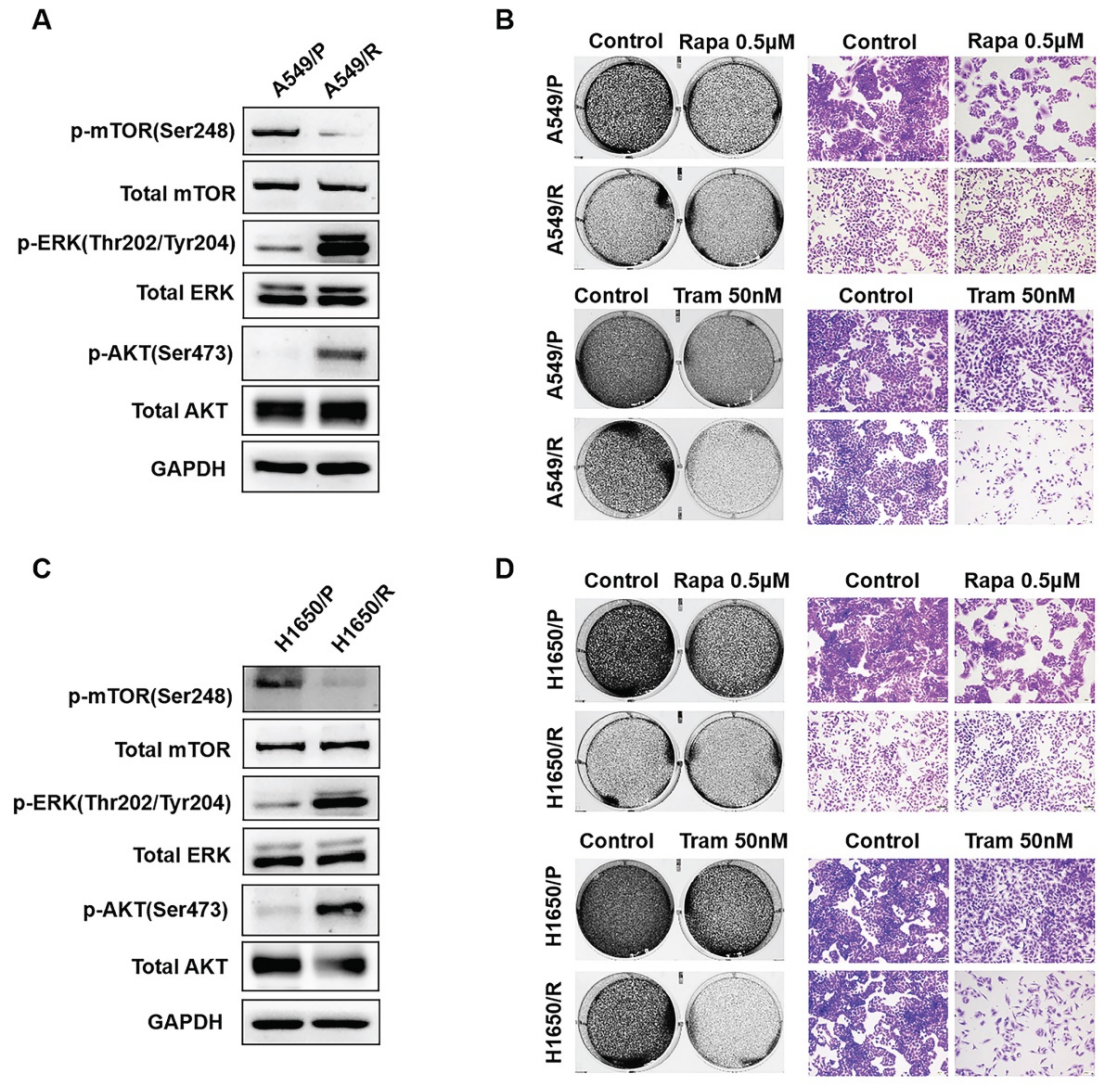
**ERK inhibition circumvents resistance to mTOR blockade. (A, C)** Rapamycin-resistant A549 and H1650 cells were generated by exposing the parental cells to rapamycin (4μM) for continuous 3 months, and the expressions of p-AKT, AKT, p-mTOR, mTOR, p-ERK, ERK were determined by western blots. **(B, D)** The parental or rapamycin-resistant NSCLC cells were treated by 500 nM rapamycin or 50 nM trametinib, and cells were stained by crystal violet.

Although mTOR inhibitors have therapeutic responses in many types of cancer, drug resistance inevitably develops due to activation of compensatory mechanisms 20. To identify determinants of rapamycin resistance, A549 and H1650 cells were exposed to high concentrations of rapamycin (4μM) for 3 months to acquire resistant colonies. In both rapamycin-resistant A549 and H1650 cells, p-ERK was highly expressed while total ERK showed no obvious change, suggesting that ERK activation might render rapamycin resistance (Fig. 1a, 1c). Not surprisingly, rapamycin-resistant NSCLC cells were more exquisitely sensitive to single-dose of trametinib treatment than its parental cells (Fig. 1b, 1d), indicating that activated ERK strongly contributed to rapamycin resistance. To determine whether, in turn, trametinib-resistant cells are sensitive to rapamycin, we treated trametinib-resistant cells or their parental cells with rapamycin. Interestingly, trametinib-resistant A549 and H1650 cells showed increased remarkable sensitivity to rapamycin treatment when compared with the parental cells (Fig. S1a). Thus, these results implicate that activation of ERK was responsible for rapamycin resistance in NSCLC cells, and ERKi was sufficient to overcome resistance.

### ERKi has a synergistic effect with rapamycin to inhibit proliferation in NSCLC cells

**Figure 2:**
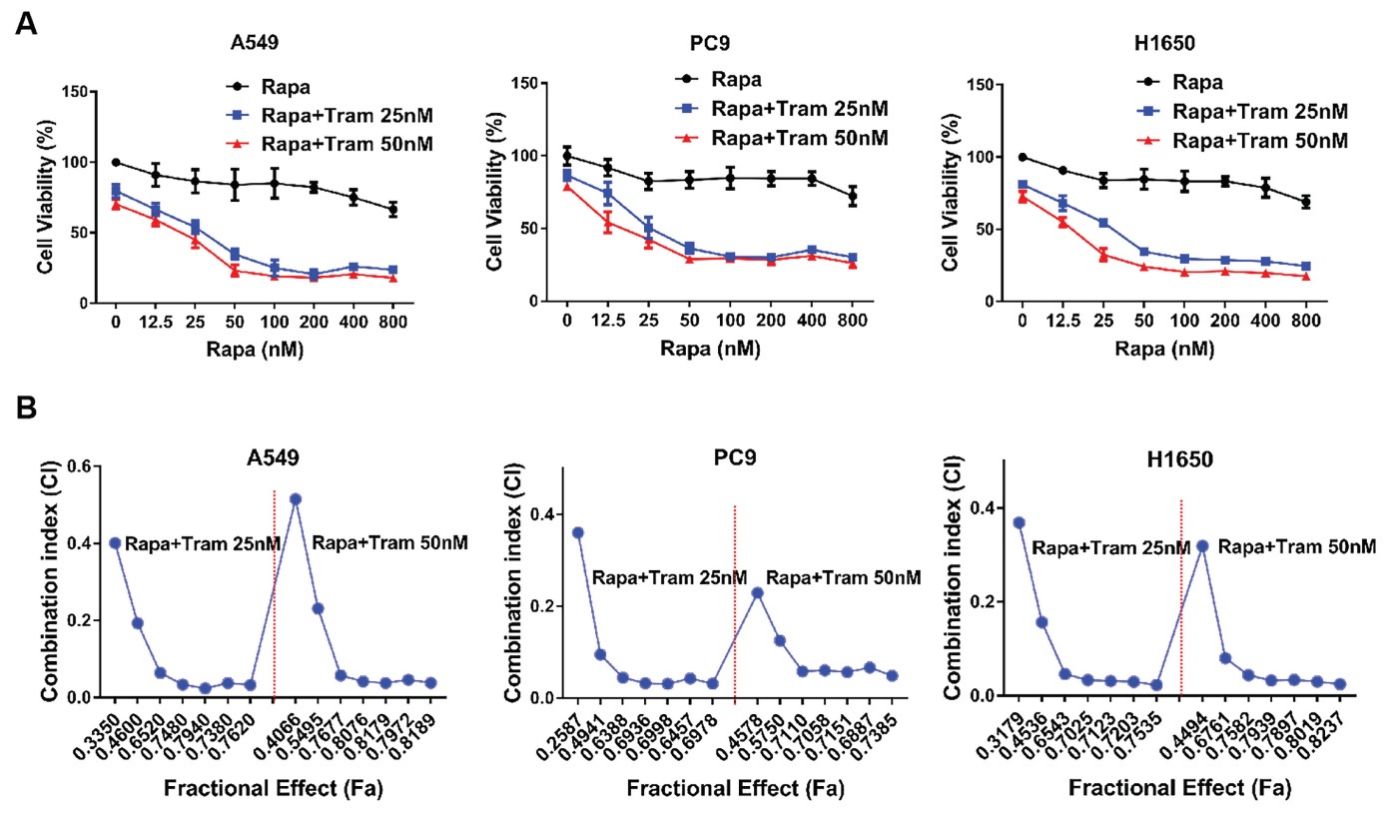
** ERKi has a synergistic anti-cancer effect with rapamycin. (A)** A549, PC9 and H1650 cells were treated with increasing concentrations of rapamycin alone, or 25, 50 nM trametinib, or in combination for 48h, and cell viability was measured by CCK8 assay. **(B)** CCK8 data were analyzed by CompuSyn software to calculate CI between trametinib and rapamycin at different concentrations in A549, PC9 and H1650 cells, and Fa-CI plot was shown.

Drug combination has been increasingly applied as a strategy to enhance the efficacy of anti-tumor treatment. Since rapamycin-resistant NSCLC cells were more sensitive to trametinib as shown in above experiments, we therefore investigated whether there exists a synergistic anti-tumor effect between rapamycin and trametinib. As displayed in Fig. 2a, when increasing concentration of rapamycin combined with trametinib (25 nM or 50 nM), the viability of A549, PC9 and H1650 cells was decreased compared with rapamycin alone treatment. To identify synergistic two-drug combination, data were analyzed using CompuSyn to calculate CI 21, 22. Using this approach, CI values > 1 indicates antagonistic, 0.9 < CI values < 1 indicates additive, CI values < 0.9 indicates synergistic. Based on CI values, as expected, combination of rapamycin with trametinib resulted in clearly synergistic interactions (CI value: 0.02-0.51) in A549, PC9 and H1650 cells (Fig. 2b).

**Figure 3:**
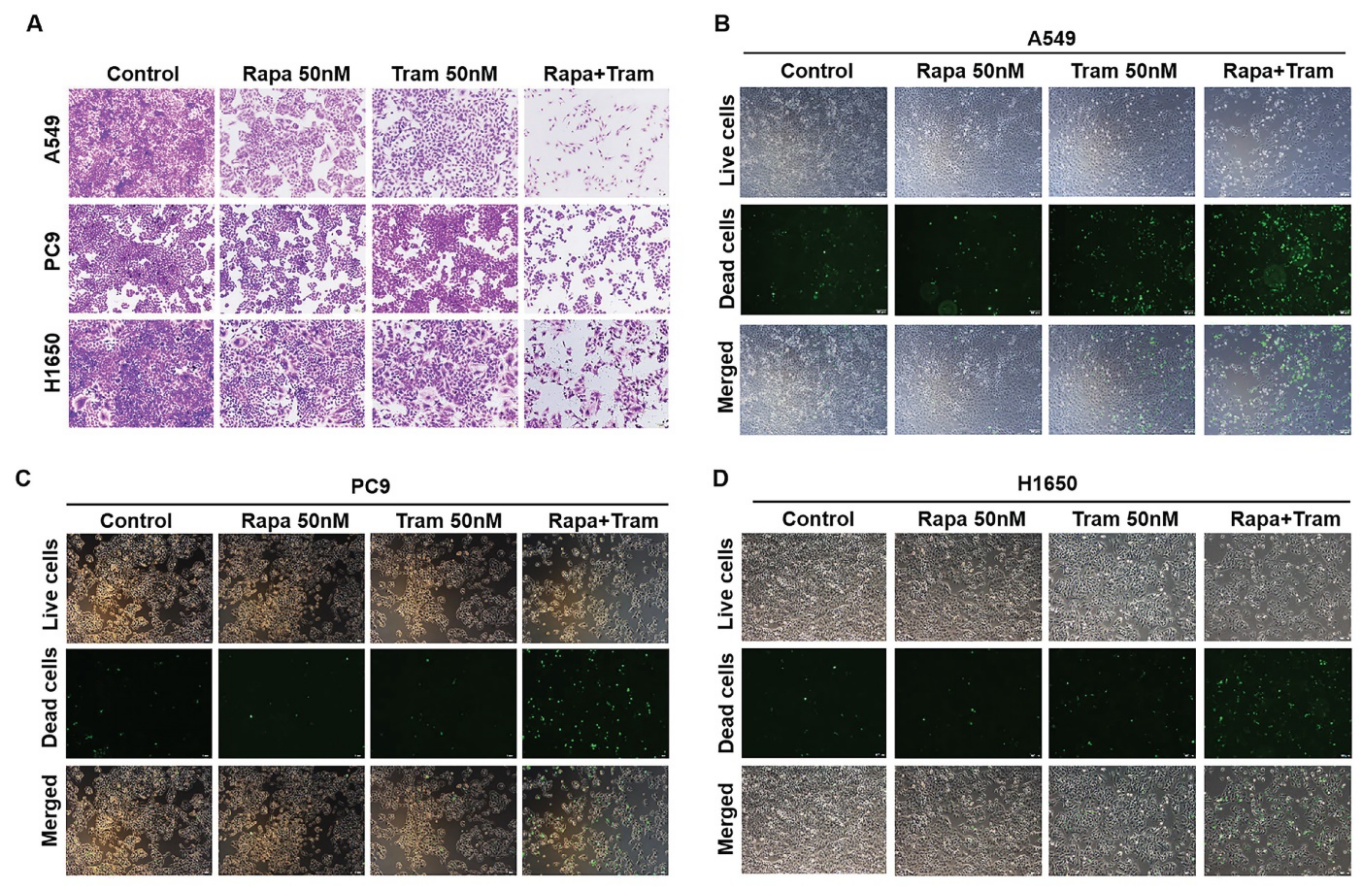
**Trametinib combined with rapamycin induces cell death in NSCLC cells. (A)** A549, PC9 and H1650 cells were treated with 50 nM rapamycin alone, or 50 nM trametinib alone, or in combination for 48 hours, and cells were stained by crystal violet.** (B-D)** A549, PC9 and H1650 cells were treated with 50 nM rapamycin alone, or 50 nM trametinib alone, or in combination for 24 hours, and the dead cells were detected by SYTOX Green staining.

To visualize the reliability of synergy between rapamycin and trametinib, we used crystal violet staining to assess the anti-proliferative effect of the combined treatments on NSCLC cells. As illustrated in Fig. 3a, rapamycin or trametinib alone could reduce cell density of NSCLC cells compared with the control. Notably, rapamycin combined with trametinib synergistically decreased the number of cells, compared to either single agent in A549, PC9 and H1650 cells (Fig. 3a). Similar results were also observed with combination of rapamycin and another ERKi, MEK162, indicating that ERKi can synergize with rapamycin to inhibit the proliferation of NSCLC cells (Fig. S1b). To confirm whether other mTORi have the similar synergy with trametinib, we tested torin1 and OSI-027 (mTORi) to combine with trametinib. As expected, torin1 or OSI-027 with trametinib also yielded a potent synergistic effect on proliferation of NSCLC cells (Fig. S2a, S2b). In addition, this combinational treatment was also applied to hepatocellular carcinoma cells (Fig. S2c) and similar results were obtained. To assess the synergetic effect of rapamycin and trametinib on cell death, SYTOX Green was used to stain dead cells after treatments. The number of dead cells was remarkably increased after combination treatment of rapamycin with trametinib when compared to either alone in A549, PC9 and H650 cells (Fig. 3b-3d). Together, these data indicate that dual inhibition of ERK and mTOR pathways yielded a synergetic anti-tumor activity in NSCLC cells.

### Trametinib synergized with rapamycin to induce G1-phase arrest and promote cell apoptosis in NSCLC cells

**Figure 4:**
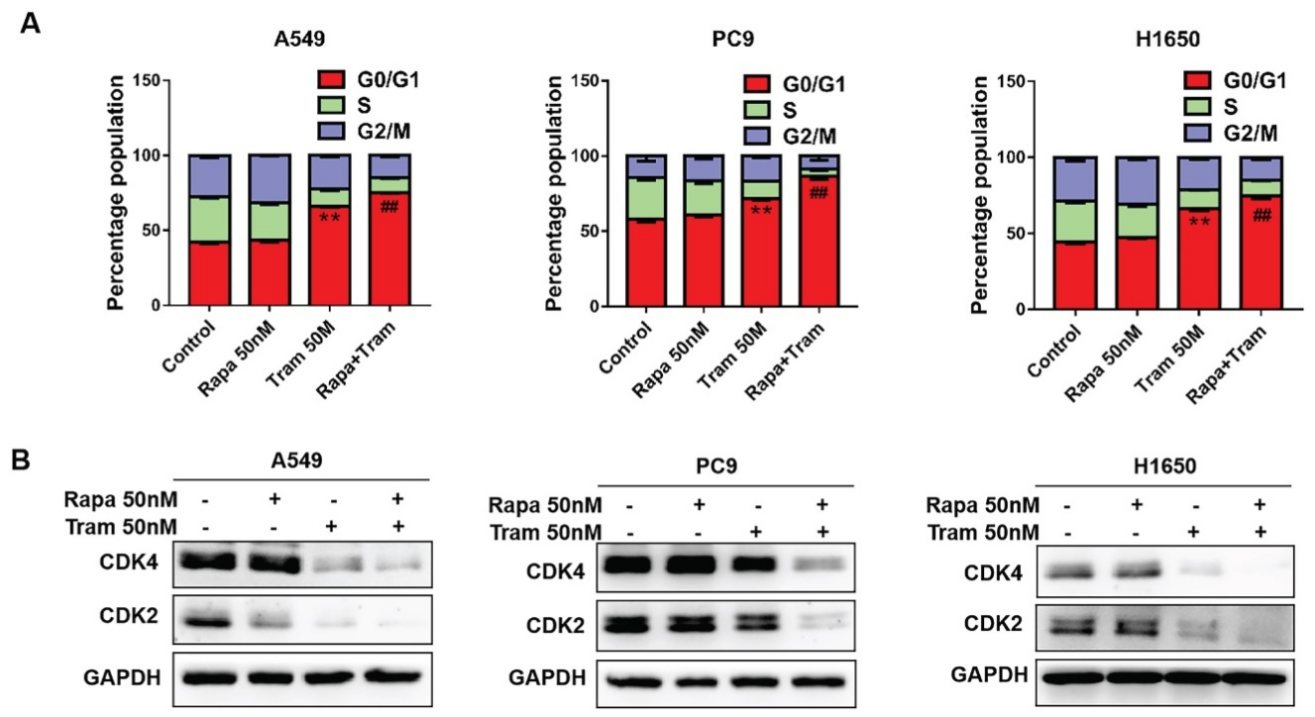
**Trametinib synergizes with rapamycin to induce cycle arrest of NSCLC cells in the G1-phase. (A)** A549, PC9 and H1650 cells were treated with 50 nM rapamycin alone, or 50 nM trametinib alone, or in combination for 48 hours, and the cell cycle distribution was measured by flow cytometry. **(B)** Western blots of CDK2 and CDK4 in NSCLC cells treated with 50 nM rapamycin alone, or 50 nM trametinib alone, or in combination for 48 hours. Representative data were shown from three independent replicates. Error bars represent mean ± SD, **P < 0.01. vs control,^ ##^P < 0.01. vs rapamycin or trametinib alone.

In a cell division, it must complete the cell cycle 23. To determine if combination of rapamycin and trametinib affects the cell cycle of NSCLC cells, we examined the cell cycle profile by flow cytometry. As shown in Fig. 4a, the percentage of cells arrested at G1-phase was increased after trametinib treatment, while rapamycin alone almost had no effect on cell cycle. Notably, trametinib synergized with rapamycin to induce a stronger accumulation of cells in G1-phase, compared with rapamycin or trametinib alone. It has been reported that cyclin-dependent kinases 2/4 (CDK2/4) specifically regulate cellular transition from the G1 to S-phase 24. Here, we found that CDK2 and CDK4 expressions were reduced in A549 and H1650 cells treated by trametinib alone or in combination with rapamycin, whereas no significant changes appeared in rapamycin-treated cells (Fig. 4b). And, in PC9 cells, trametinib treatment induced moderate decrease in CDK4 and CDK4, but combination of rapamycin and trametinib resulted in a dramatic reduction of CDK2 and CDK4 levels (Fig. 4b).

**Figure 5:**
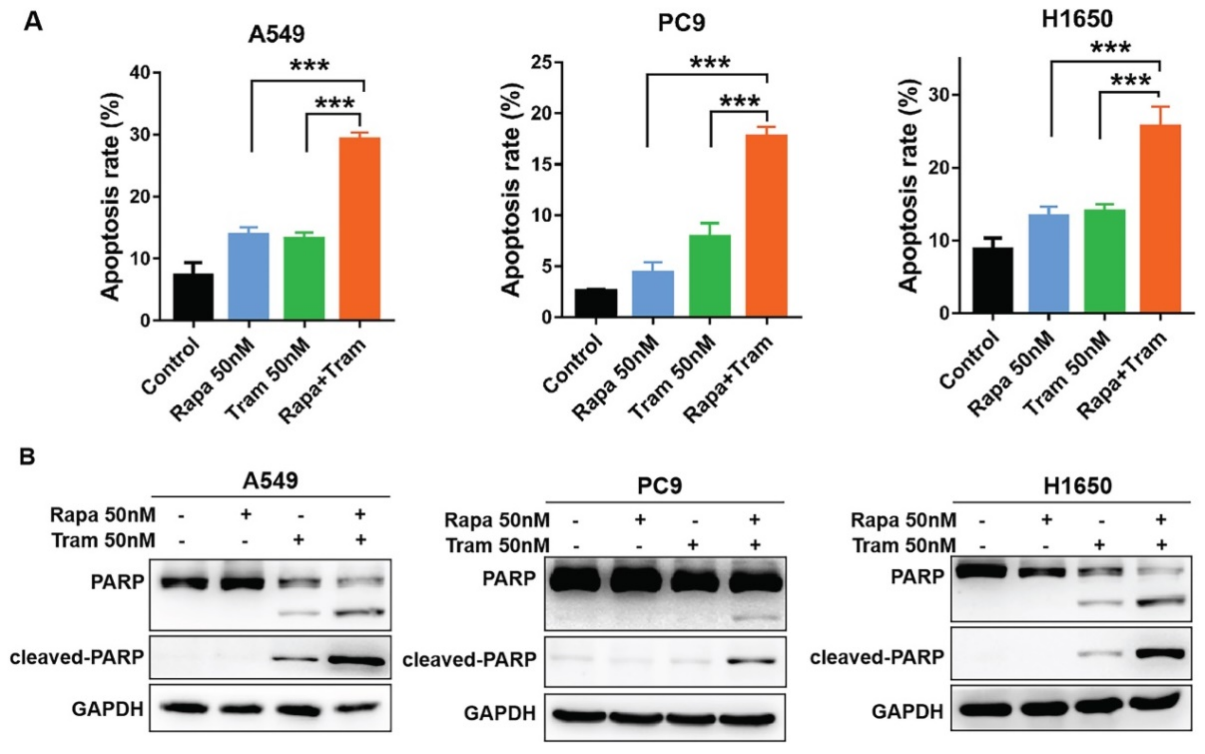
**Trametinib synergized with rapamycin to promote apoptosis in NSCLC cells. (A)** A549, PC9 and H1650 cells were treated with 50 nM rapamycin alone, or 50 nM trametinib alone, or in combination for 48 hours, and the apoptotic cells were measured by flow cytometry analysis.** (B)** Western blot analyses of PARP and cleaved PARP in NSCLC cells treated with 50 nM rapamycin alone, or trametinib alone, or in combination for 48 hours. Representative data were shown from three independent replicates. Error bars represent mean ± SD, ***P < 0.001.

To further characterize the cellular phenotype in response to the combined drug treatment, we employed flow cytometry to detect cell apoptosis. Indeed, increased apoptotic cells were observed following the treatment of either single agent, compared to untreated cells (Fig. 5a). Of note, rapamycin plus trametinib caused a striking increase in the percentage of apoptotic cells in NSCLC cells when compared to the single-agent treatment (Fig. 5a). Given that cleavage of PARP is required in the process of apoptosis, cleaved PARP has been considered as an excellent hallmark of apoptosis 25. Western blots data for PARP and cleaved PARP showed that 50 nM trametinib, rather than 50 nM rapamycin, increased the level of cleaved PARP in H1650 and A549 cells, while rapamycin in combination with trametinib resulted in more pronounced increase in cleaved PARP level (Fig. 5b). Interestingly, neither rapamycin nor trametinib had an obvious effect on the level of cleaved PARP in PC9 cells, whereas rapamycin combined with trametinib significantly upregulated the cleaved PARP (Fig. 5b). Thus, these results suggest that NSCLC cells were highly sensitive to the combined treatment of rapamycin and trametinib in large part by induction of G1-phase cycle arrest and apoptosis.

### Combined treatment of rapamycin and trametinib suppressed AKT, ERK, mTOR and 4EBP1 signaling

**Figure 6:**
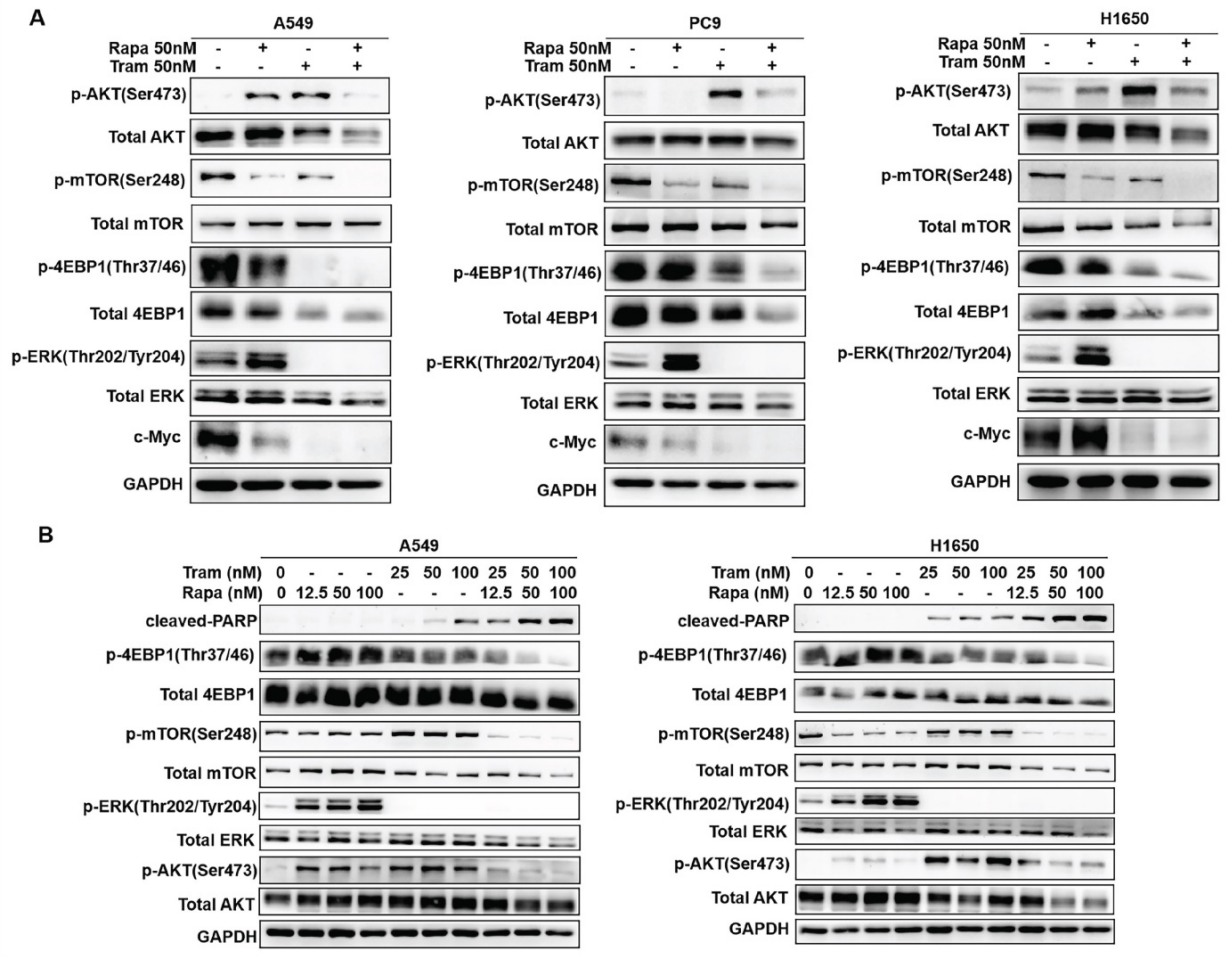
**Combined treatment of rapamycin and trametinib suppressed AKT, mTOR and 4EBP1 pathways. (A)** A549, PC9 and H1650 cells were treated with 50 nM rapamycin alone, or 50 nM trametinib alone, or in combination for 48 hours, and the expressions of p-AKT, AKT, p-mTOR, mTOR, p-4EBP1, 4EBP1, p-ERK, ERK and c-myc were determined by western blots.** (B)** A549 and H1650 cells were treated with 25, 50, 100 nM rapamycin alone, or 25, 50, 100 nM trametinib alone, or in combination, the indicated proteins were detected by western blots. Representative data were shown from three independent replicates.

**Figure 7:**
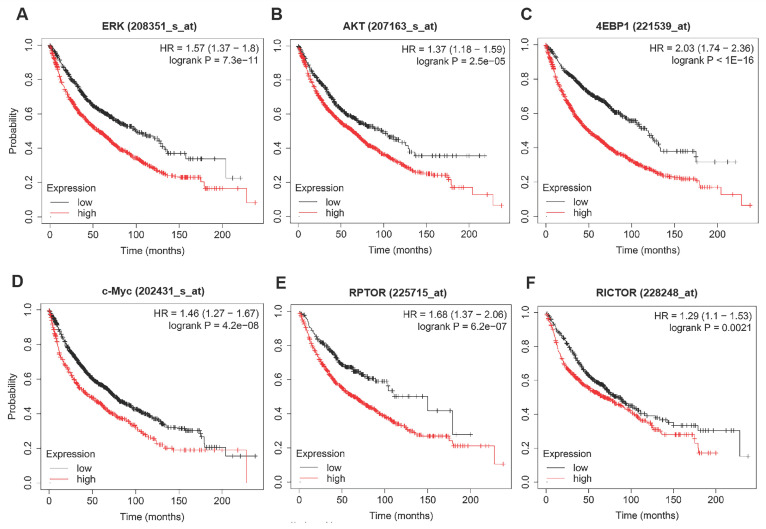
** The prognostic values of ERK, AKT, c-myc, mTOR and 4EBP1 in lung cancer patients. (A-F)** The survival curves comparing lung cancer patients with high (red) and low (black) genes expression (ERK, AKT, c-myc, 4EBP1, RPTOR and RICTOR) were plotted by the Kaplan-Meier plotter. *P* value < 0.05 was considered as statistically significant difference.

To discover the possible mechanisms by which rapamycin and trametinib yielded a dramatically synergistic anti-tumor activity, we performed western blots to test the protein profiles of the cell signaling pathways regulated by the two agents in NSCLC cells. Consistent with Fig. 1a, 1c, single-dose rapamycin-treated NSCLC cells showed significant upregulation of p-ERK, and rapamycin-stimulated activation of ERK was completely abrogated by trametinib (Fig. 6a). Similarly, treatment with trametinib alone or in combination with rapamycin led to a complete inactivation of oncogene, c-myc in NSCLC cells (Fig. 6a). In addition, A549 and H1650 cells showed significant increase of p-AKT in response to either rapamycin or trametinib alone, whereas combination of rapamycin and trametinib failed to activate AKT (Fig. 6a). In PC9 cells, only trametinib alone triggered activation of AKT, and elevated p-AKT was substantially restored in the presence of rapamycin (Fig. 6a). Indeed, rapamycin treatment led to significant inhibition of p-mTOR in NSCLC cells, and trametinib in combination with rapamycin highly suppressed mTOR pathway when compared with single-agents (Fig. 6a). Interestingly, trametinib or plus rapamycin caused a dramatic reduction in p-4EBP1 and total 4EBP1, whereas rapamycin alone had no such effect (Fig. 6a). To confirm these data independently, we employed different concentrations of rapamycin or trametinib alone, or in combination to repeat that experiment. And, as shown in Fig. 6b, the results were largely consistent with the observations in Fig. 6a and these signaling proteins were rapidly decreased with increasing concentration of the two agents. In addition, we performed the Kaplan-Meier (KM) plotter to assess the prognostic significance of ERK, AKT, c-myc, mTOR and 4EBP1 in lung cancer patients. As expected, KM plotter analysis showed that lung cancer patients who highly expressed ERK, AKT, c-myc, 4EBP1, RPTOR (regulatory associated protein of mTOR complex 1) and RICTOR (RPTOR independent companion of mTOR complex 2) has a shorter survival time (Fig.7). Taken together, we conclude that rapamycin in combination with trametinib suppressed AKT, ERK, mTOR and 4EBP1 pathways in NSCLC cells.

### Co-administration of rapamycin and trametinib inhibits tumor growth in *in vivo* model

**Figure 8:**
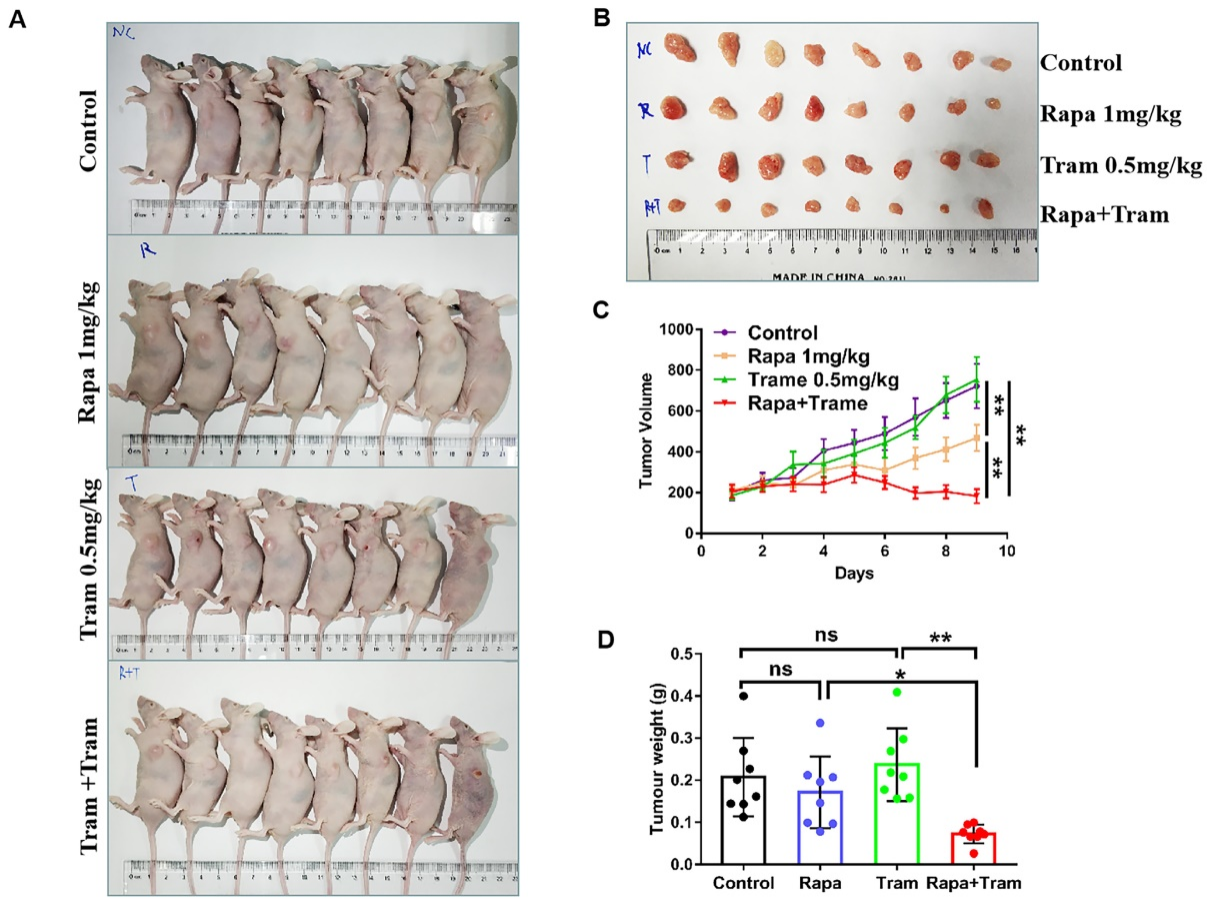
** Co-administration of rapamycin and trametinib inhibits tumor growth in mouse model.** PC9 cells were subcutaneously injected into the left flank of mice. Mice were treated with the vehicle; rapamycin (1mg/kg, i.p.); trametinib (0.5mg/kg, i.g.); rapamycin + trametinib, for continuous 9 days. **(A)** After treatment, mice were humanely euthanized. Photographs of mice were shown (n=8). **(B)** Photographs of tumors dissected out mice at each treatment group (n=8).** (C)** Tumor volumes were measured every day, and growth curves of tumors were shown. **(D)** Tumor weight was measured. Error bars represent mean ± SD, *P < 0.05, **P < 0.01, ns, no significant difference.

**Figure 9:**
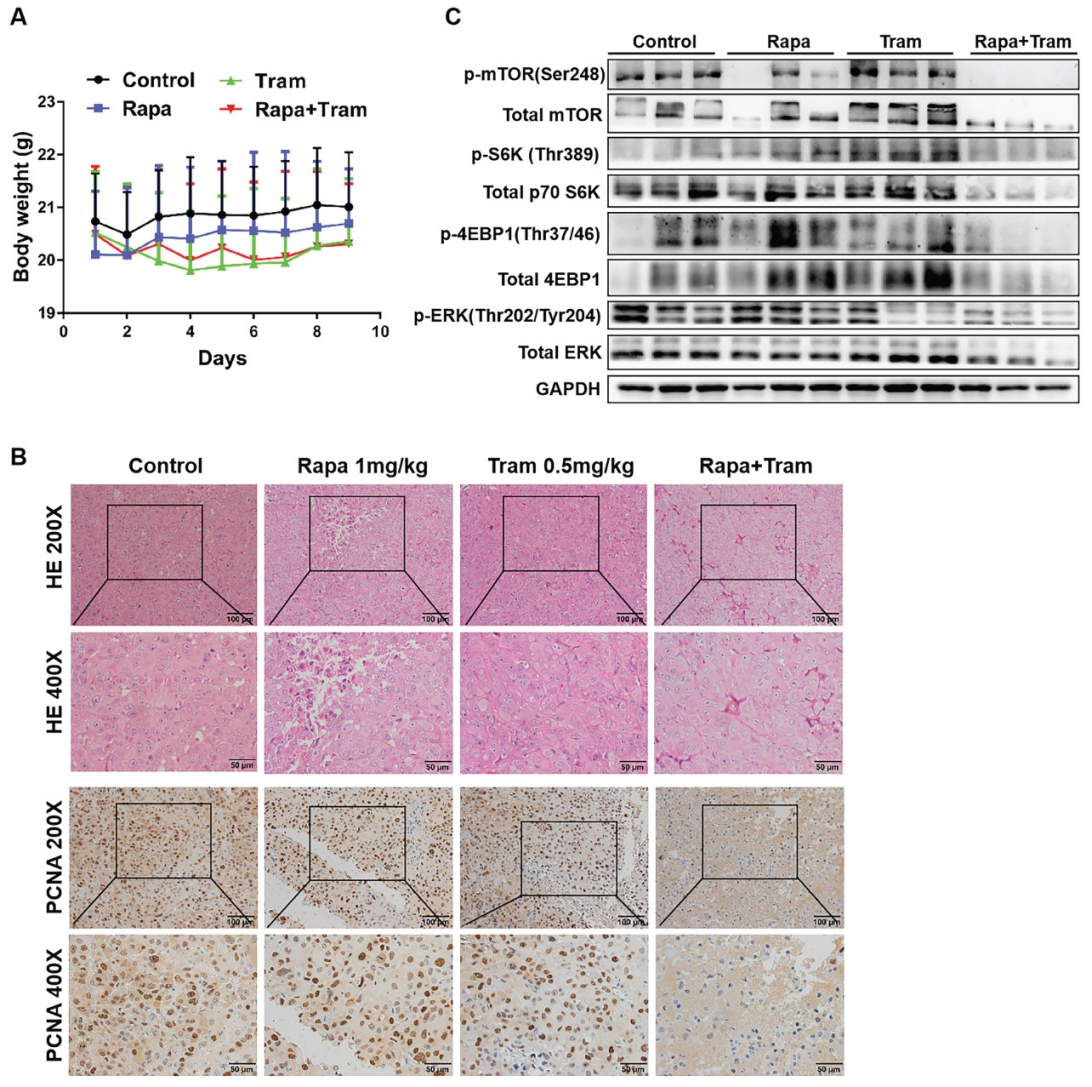
**Co-administration of rapamycin and trametinib inhibits tumor growth in mouse model. (A)** Mouse weight of each treatment group was measured every day. **(B)** The expressions of indicated protein in mice tumor were detected by western blots. **(C)** Representative images of hematoxylin-eosin staining and immunohistochemical staining of PCNA of tumors.

Since *in vitro* experiments cannot reflect the interactions between tumor cells and drugs in the whole body, we investigated the synergetic activity of rapamycin and trametinib in the xenograft tumors. Mice bearing established tumors were treated with the vehicle, 1mg/kg rapamycin 26, 0.5mg/kg trametinib 27, or in combination. As expected, after continuous 9-day treatment, the tumor growth curve of trametinib group was not divergent from the vehicle group, indicating that 0.5mg/kg trametinib has not effect on NSCLC xenograft tumors, while single-agent rapamycin signficantly reduced tumor growth as compared to the control group (Fig.8c). Importantly, the combination of rapamycin and trametinib resulted in a striking overall reduction in the tumor weight and volume,when compared to the monotherapy (Fig. 8a-8d). This was confirmed by the immunohistochemical results that co-administration of rapamycin and trametinib reduced the number of PCNA-positive tumor cells compared with the single-agents treatment (Fig. 9b). In addition, no considerable changes in mouse weight were observed in single-agent or the combination-treated mice at the end of the experiment (Fig. 9a). We also detected the related protein expressions in tumors, and found that the levels of both phosphorylated mTOR, S6K, 4EBP1 and ERK in mice co-treated with rapamycin and trametinib were lower than the monotherapy or the control (Fig. 9c). Overall, these data demonstrate that combined treatment of rapamycin and trametinib obviously inhibits the established tumors in mouse model.

## Discussion

Our study identifies a superior two-drugs combined strategy for treatment of NSCLC. Since rapamycin has limited activity against established tumors, effective strategies to enhance the anti-tumor effect of rapamycin are urgently needed. In this regard, ERKi synergized with rapamycin to suppress tumor growth *in vitro* and *in vivo* . First, ERK pathway was activated in rapamycin-resistant NSCLC cells, and resistant cells were susceptible to trametinib. Second, both rapamycin and trametinib caused activation of AKT, and combination of rapamycin and trametinib prevented such reactivation of AKT. Third, combined treatment of rapamycin and trametinib inhibited mTOR and 4EBP1 pathways. Thus, rapamycin in combination with ERKi is an effective and reasonable approach for treatment of NSCLC. Interestingly, rapamycin combined with trametinib was reported to promote longevity in *Drosophila* 28.

mTOR is an essential integrator of cell growth, apoptosis, and metabolism 29. It forms two distinct complexes, called mTOR complex 1 (mTORC1) and mTORC2, of which only mTORC1 is sensitive to the macrolide rapamycin 30-32. Specifically, mTORC1 can phosphorylate ribosomal protein S6 kinase (S6K) and eIF4E binding protein 1 (4EBP1) to increase translation of mRNAs, while mTORC2 controls cell survival via activation AKT and SGK1 pathway 33, 34. Initial studies mainly focus on inhibition of mTORC1 with rapamycin. However, incomplete inhibition of mTORC1 by rapamycin is often observed, by which rapamycin doesn't affect mTOR effector 4EBP-1 5. In addition, rapamycin and its analogues treatment lead to the feedback activation of AKT pathway 35. Thus, the acquired drug resistance to rapamycin inevitably develops. Here, we found that rapamycin did not inhibit 4EBP1, but strongly activated AKT pathway in NSCLC cells. Importantly, combination of rapamycin with trametinib abolished AKT signaling, and resulted in a dramatic inactivation of 4EBP1. Thus, combined treatment counteracted rapamycin-stimulated activation of AKT and did inhibit 4EBP1 that rapamycin was supposed to do.

Rapamycin and its analogues have disappointing clinical results, mainly because tumor cells cause a complex resistant mechanism in response to rapamycin. It has been reported that inhibition of mTOR also results in a compensatory and redundant activation of ERK signaling, and such ERK activation is considered as pro-survival process for tumor cells 36, 37. We thus employed ERKi, trametinib and MEK162 to combine with rapamycin for the treatment of NSCLC cells. As expected, a high level of p-ERK was detected in single-dose or continuous rapamycin-treated NSCLC cells. In contrast, rapamycin-induced ERK activation was completely abrogated in the presence of trametinib. Accordingly, ERKi sensitized NSCLC cells to rapamycin treatment. Thus, rapamycin-stimulated activation of ERK conferred drug resistance to rapamycin. Another important mechanism for this synergy is that dual inhibition of mTOR and ERK resulted in a maximal enhancement of apoptosis and G1-phase arrest.

Since RAS/ERK pathway is aberrant activated in many human cancers, a number of specific ERKi display potent clinical efficacy in the treatment of BRAF-mutant melanoma 38, 39. Despite this, the resistance of ERKi emerges as an intractable problem, and its complex feedback loops and tight crosstalks with other pathways contribute to its resistant prototype 40, 41. It has been clear that ERK has extensive crosstalk with AKT pathway, and such crosstalk reduces the effect of single inhibition 42. Trametinib and PD0325901, for example, induces AKT activation, and blockage of AKT pathway overcomes resistance of ERKi 43. As such, crosstalk between AKT and ERK pathway is a rational basis for combination therapies in cancer 44. This notion was confirmed in our work. Blocking ERK signaling by trametinib treatment caused a paradoxical activation of AKT. Interestingly, rapamycin alone induced AKT activation, but combined with trametinib to inhibit AKT pathway. This might explain why trametinib-resistant cells were susceptive to rapamycin treatment. It appears that activation of AKT was associated with rapamycin and trametinib resistance, and combined rapamycin with trametinib could circumvent this tricky problem. Thus, mTORi in combination with ERKi has therapeutic benefit for NSCLC cells.

## Conclusions

Given the complex nature of cancer, it is unlikely that a single compound can be the most effective therapy. Our data provide a strong rationale for combining rapamycin with trametinib for treatment of NSCLC. Notably, *in vivo* model demonstrated that trametinib could maximize the anti-cancer efficiency of rapamycin, with minimal drug toxicity. Thus, these data support further evaluation of combination of rapamycin and trametinib in clinical trials. Nevertheless, our work has a few limitations. First, it is not clear that whether trametinib alone or in combination with rapamycin induces G1-phase arrest by regulation of ERK/c-myc pathway. Second, the detailed effect of 4EBP1 in synergy between rapamycin and trametinib is also unclear.

## Supplementary Material

Supplementary figures.

## Abbreviations

NSCLC: non-small cell lung cancer
mTOR: mammalian target of rapamycin
mTORi: mTOR inhibitors
ERK: extracellular regulated protein kinase
ERKi: ERK inhibitors
FDA: food and drug administration
BRAF: B-Raf proto-oncogene, serine/threonine kinase
CI: combination index
CDK2/4: cyclin-dependent kinases 2/4
PARP: poly ADP-ribose polymerase
PCNA: proliferating cell nuclear antigen
S6K: ribosomal protein S6 kinase
4EBP1: eIF4E binding protein 1
mTORC1: mTOR complex 1
mTORC2: mTOR complex 2
RPTOR: regulatory associated protein of MTOR complex 1
RICTOR: RPTOR independent companion of MTOR complex 2
SGK1: serum/glucocorticoid regulated kinase 1
