# Supplementary figures and images for: Rapamycin and trametinib: a rational combination for treatment of NSCLC
Source: Int J Biol Sci. 2021 Jul 25;17(12):3211–23. doi: 10.7150/ijbs.62752 (PMC8375233; doi:10.7150/ijbs.62752)

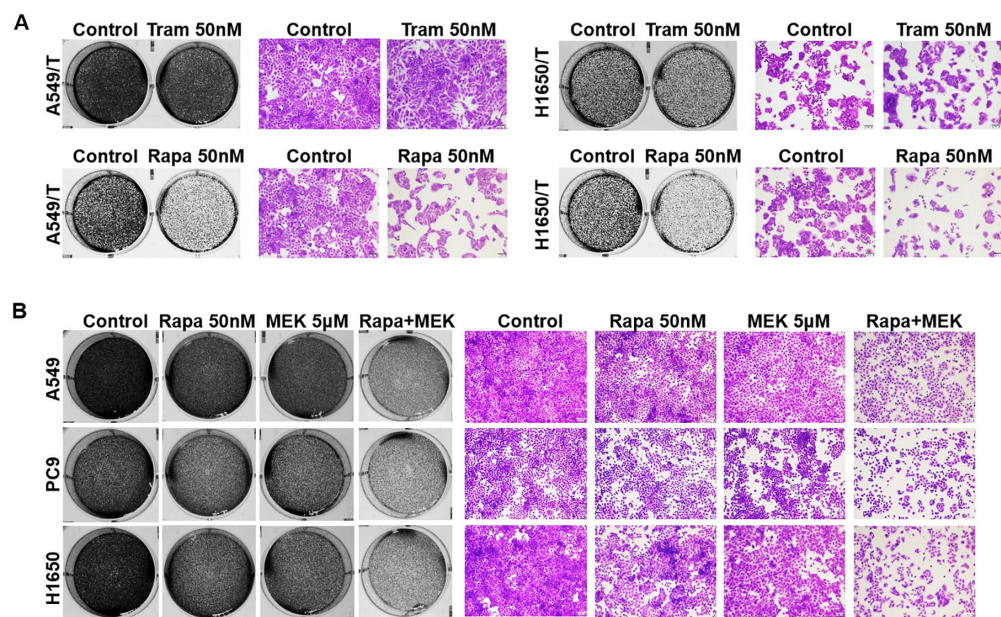

Supplementary figure. 1

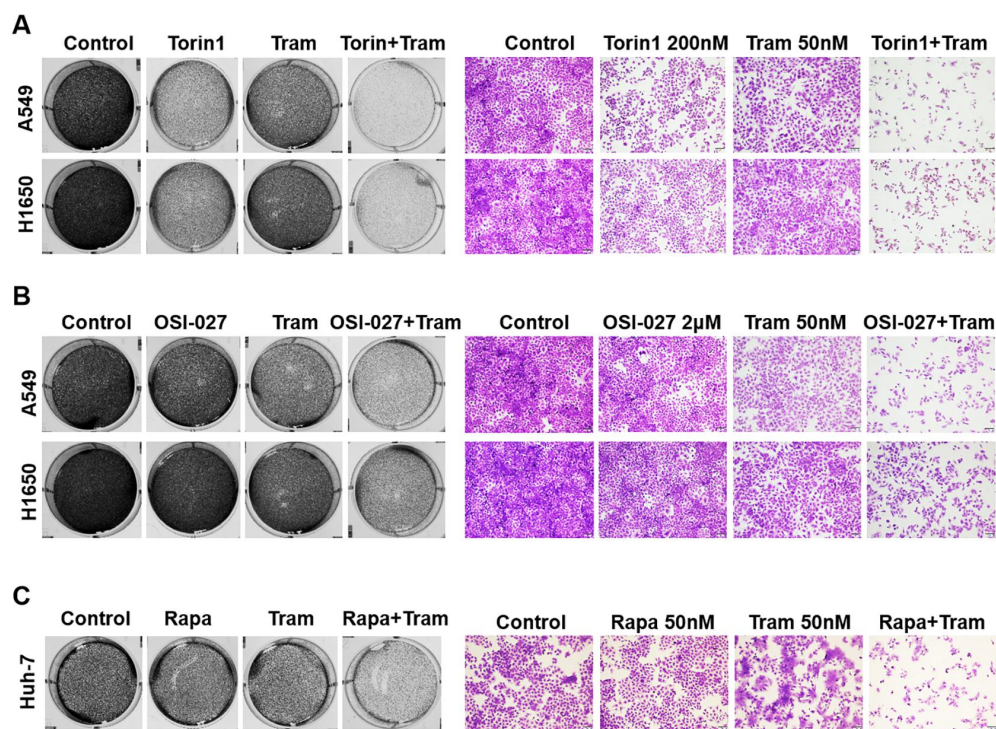

Supplementary figure. 2

Supplement: Supplementary file 1 — Supplementary figures. [file ijbsv17p3211s1.pdf]
